# Comparison between the World Health Organization and Bahraini children growth standards

**DOI:** 10.1590/1984-0462/2023/41/2022050

**Published:** 2023-05-29

**Authors:** Shahzalan Almughlaq, Abdel-Ameer Al-Laith, Salwa Al-Thawadi

**Affiliations:** a University of Bahrain, Al Sakhir, Kingdom of Bahrain

**Keywords:** Growth charts, Bahrain, Weight, Length, Body mass index, Curvas de crescimento, Barein, Peso, Comprimento, Índice de massa corporal

## Abstract

**Objective:**

The aim of this study was to investigate the growth patterns of Bahraini female and male infants/young children aged 0–24 months in the Kingdom of Bahrain.

**Methods:**

A cross-sectional approach was employed to track the growth parameters among healthy Bahraini female and male infants/children aged 0–24 months. A multistage probability sampling criteria was used to collect information from official records. Anthropometric measurements (weight and length) and demographic characteristics on feeding practices were gathered. Generalized Additive Models for Location Scale and Shape (GAMLSS)/Lambda-Mu-Sigma methodology was implemented to select distribution type, optimize smoothing parameters, perform regression of growth models, and construct percentiles and Z-score charts and tables for weight for age, length for age, length for weight, and body mass index (BMI) for age.

**Results:**

Findings were compared with WHO Multicentre Growth Reference Study (MGRS) data. A total of 403 healthy infants/children (210 males and 193 females) were recruited. At birth, the mean weight, length, and BMI were 3.2±0.4 kg, 3.1±0.4 kg, 49.7±2.3 cm, 48.8±2.1 cm, 13.2±1.6 kg/m^2^, and 12.8±1.5 kg/m^2^ for males and females, respectively. Anthropometrics of males were all statistically significantly higher than those of females at all age levels. The length and weight of the Bahraini infants/children were slightly higher than those of the WHO-MGRS.

**Conclusion:**

The outcomes of this study, presented as charts and tables, showed significant differences in comparison with the WHO-MGRS reference charts. Specifically, Bahraini children aged between 0 and 24 months of both sexes were taller and heavier than their cohorts in the MGRS reference charts. Further longitudinal studies are needed for monitoring the growth pattern of children using body composition methods, adiposity markers, and determinant factors of growth to investigate this deviation from the WHO-MGRS.

## RESUMO

**Objetivo:**

Investigar os padrões de crescimento de lactentes de 0–24 meses dos sexos feminino e masculino, no Reino do Bahrein.

**Métodos:**

Estudo transversal de acompanhamento de lactentes saudáveis com 0–24 meses de idade dos sexos feminino e masculino, em Bahrein. A amostragem probabilística de estágios múltiplos foi utilizada para coletar informações dos registros oficiais. Medidas antropométricas (peso, comprimento), práticas alimentares e variáveis demográficas foram coletadas. A metodologia *Generalized Additive Models for Location Scale and Shape* (GAMLSS)/*Lambda-Mu-Sigma* (LMS) foi utilizada para selecionar o tipo de distribuição dos parâmetros antropométricos, otimizar os parâmetros de suavização, para fazer a análise de regressão de modelos de crescimento e para construir percentis e gráficos e tabelas de escore Z para peso para idade, comprimento por idade, comprimento por peso e índice de massa corporal (IMC) para idade. Os resultados foram comparados com os dados do Estudo Multicêntrico de Referência de Crescimento da Organização Mundial da Saúde (OMS).

**Resultados:**

O total de 403 lactentes (210 do sexo masculino e 193 do feminino) foi incluído no estudo. Ao nascimento, a média de peso, comprimento e IMC foi de 3,2±0,4 kg, 3,1±0,4 kg, 49,7±2,3 cm, 48,8±2,1 cm, 13,2±1,6 kg/m2 e 12,8±1,5 kg/m2 para o sexo masculino e para o feminino, respectivamente. Todos os índices antropométricos foram maiores no sexo masculino. O comprimento e o peso dos lactentes em Bahrein foram ligeiramente superiores aos da OMS.

**Conclusões:**

Os lactentes do Bahrein com idade entre 0 e 24 meses de ambos os sexos eram mais altos e mais pesados do que os lactentes analisados na coorte da OMS. Mais estudos longitudinais são necessários para monitorar o padrão de crescimento de crianças usando métodos de composição corporal, marcadores de adiposidade e fatores determinantes do crescimento para investigar esse desvio dos padrões da OMS.

## INTRODUCTION

On a global scale and within the primary health systems, almost all countries have a constitutionally and morally binding responsibility to care for the health of newborns.^1^ Countries strive to meet the growing needs to achieve national plans and goals. Monitoring of growth indicators or anthropometric parameters, such as weight, height, head circumference, and skin folds, are among these concerns.^2^ Furthermore, regular monitoring of some of these parameters, from birth to adolescence, is an essential part of evaluating national health plans and learning about the quality of life and the health of individuals.^3^

Despite the existence of internationally recognized and widely implemented guidelines and recommendations set by the World Health Organization (WHO) in the form of the International Growth Charts,^4^ many countries have experienced the inadequacy of the WHO standards and references and established their own national growth curves.^5^ The WHO child growth standards (0–5 years) are based on the Multinational Growth Reference Study (MGRS), which monitored children growing under conditions of health and nutritional suitability, which may not ensure that it will provide the same degree of statistical fitness for each.^6^ Furthermore, the main limitation of the WHO growth charts is that they do not consider the variations in patterns of growth among different human populations. These variations are attributable to genetic, nutritional, environmental, cultural, economic, or other differences.^7^ In fact, many recent studies have shown that growth patterns of local children differ from the international mean standards.^8,9^ It is claimed that using non-population-specific growth charts may have various consequences, such as the misclassification of perfectly healthy and developing children as malnourished.^10,11^

The 2006-WHO-MGRS growth standards are based on the Z-scores, which represent the anthropometric measurements, such as height, weight, and body mass index (BMI), as several standard deviations (SDs) below or above the reference mean or median value.^12^ Statistical estimates based on Z-score methodology are scalable and widely implemented in medicine and health-related fields. Besides their usage to monitor infants’ and children’s developmental growth, Z-scores are also used to evaluate the nutritional status of infants and children.^11^

Currently, the 2006-WHO-MGRS 0–5 years standards and growth charts are implemented in the Kingdom of Bahrain. This implementation has never been subject to verification, which implies that it is suitable for the local situation. This current study is based on the assumption that differences may be statistically significant. The proposed hypothesis of this study is that there is a significant difference between the 2006-WHO-MGRS growth charts and the actual national growth parameters (weight for age (WFA), length/height for age (LFA), height for weight (HFW), and BMI for age (BMIFA) among Bahraini infants and young children (aged 0–24 months), which warrants the development of national growth charts. The main objective of the study was to establish national physical growth standards based on a representative sample of the targeted population. Prior restrictions imposed on the sampling procedure by the local official authorities limited the scope of the study to infants/children of Bahrain nationality. Furthermore, due to space limitations, the relationship between anthropometrics and the nutritional status of the recruited subjects will not be addressed here. This study is confined to the assessment of anthropometrics and how they compare to the WHO-MGRS standards.

## METHOD

In the Kingdom of Bahrain, childbirth takes place in maternity hospitals by law. Vital data are officially and centrally recorded for follow-up until the completion of childhood. Typical recorded and monitored data include the child’s clinical history, physical development, immunizations, feeding practices, and mother’s maternity information. These data are collected during regularly scheduled visits to the child and maternity sections in the governmental health centers. Routine visits are officially scheduled at 2, 4, 6, 9, 12, 18, and 24 months after birth.

In this cross-sectional study, carried out during 2018, all the recruited subjects had already completed 24 months. The targeted population included Bahraini healthy full-term (between 37 and 42 gestational weeks) infants and children of both sexes, with birth weight greater than 2500 g and born during the year 2014.

The initial sample size was 428, and subjects who had any disease and/or health complications after delivery that could affect their growth pattern were excluded. Three subjects were excluded at the start due to noncompliance. The study design and protocol were approved by the relevant ethics committees at the University of Bahrain and the Ministry of Health in the Kingdom of Bahrain.

This study used a multistage and stratified sampling design to select a representative sample of Bahraini infants and young children. The official records of the Child and Student Health Record of the Ministry of Health (MOH) in Bahrain were used as the source of information for this study. Only limited data of interest were manually extracted and tabulated as an Excel file (Microsoft, USA). These data included weight and length/height at each specified age (birth, 2, 4, 6, 9, 12, 18, and 24 months), sex, child rank, gestational age, and mother’s age at birth.

The multistage stratified random sampling procedure to select the recruited sample was as follows: administratively, the Kingdom of Bahrain is divided into four governorates comprising five health regions with 28 official health centers distributed around the country.^13^ One health center from each health region was selected and treated as the primary sampling unit (PSU). Each PSU was sampled with a probability proportional to its population size based on the officially registered live births by health region/health center and sex. The second-stage sampling involved selecting a sex-specific sample from each health region, followed by a withdrawal of a sample from each health center proportional to the size of each sex in the third stage.

For defining the sample size (N), the following equation was used: N=Z1-α/22 p (1-p), where Z1-α/2 was the standard normal varieties (at 5% type, 1 error [p<0.05] it is 1.96, and at 1% type, 1 error [p<0.01] it is 2.58). p-values are considered significant below 0.05, hence 1.96 is used in the formula.^14^

Weight (kg) and length/height (cm) were routinely measured by attendant health professionals in each health center. The infant’s weight was measured in supine position by electronic infant scales to the nearest 0.1 g. Recumbent length was measured by a special headboard with another movable board for more accuracy to the nearest 0.1 cm. Once the child could stand, the weight and height were measured by a special digital scale with a stadiometer.

A predesigned Excel sheet (Microsoft Excel 2016) was used to collect information from the selected children’s records. The Excel sheet was designed as a table consisting of two major parts. The first part was dedicated to collecting the demographic characteristics of the child. The second part was dedicated to collecting the anthropometric data (weight, height, and BMI) and feeding practices (breastfeeding/bottle-feeding, both types, and complementary feeding) at each specified age (2, 4, 6, 9, 12, 18, and 24 months).

Outliers were detected and excluded in weight and length, if any, using Z-scores according to the WHO criteria, values with 3 SD (Z-score).^15^ Descriptive statistics of data were analyzed using SPSS (version 23.0, Chicago, IL, USA) and R programming. Prior to performing the growth modeling calculations, the data were investigated at each age level for distribution type, skewness, and kurtosis using distrplus, gamlssML functions, and the MASS and Generalized Additive Models for Location Scale and Shape (GAMLSS) packages in R. The aim of these investigations was to gain more insight when addressing distributional issues.

Frequencies and percentages were computed for the categorical variables. Mean, SD, and the 3rd, 10th, 25th, 50th, 75th, 90th, and 97th age sex-specific percentiles. Significant differences in linear growth between the MGRS and Bahrain study populations were examined by adopting the WHO cutoff value, which was considered (>0.5 SD) a benchmark for clinical significance.^12,15^

The Lambda-Mu-Sigma (LMS) method, implemented in the GAMLSS package and recommended by WHO, was used to estimate the smoothed centiles after modeling the measurement and smoothing the model parameters.^16^ At each age point, the distribution is characterized by three parameters: L (defines the skewness), M (the median), and S (the generalized coefficient of variation). Given these parameters, any centile value can be calculated using the following equations: X=M (1+LSZ) 1/L; L≠0, or X=M exp (SZ); L=0, where X is the value of the anthropometric variable and Z is the required percentile in SD units. In this method, the final growth charts are derived from smoothed percentiles rather than from the underlying distributions.

Besides accounting for the above-mentioned three smoothing parameters, GAMLSS also takes kurtosis into account as an additional smoothing parameter. In this package, the response variables (i.e., weight, height) are modeled by age, where the parameters μ, σ, ν, and τ are the modeled variables. The degrees of freedom (μ, σ, and ν) were estimated and automatically optimized using the hyperparameters and find hyper (optim) functions in the GAMLSS package, as described in Rigby and Stasinopoulos.^16^ The chosen parameters were estimated by penalized maximum likelihood. Using cubic splines smoothers, the selection was based on comparison of the generalized Akaike information criterion. GAMLSS is an extension of the generalized additive models which allows for all distribution parameters (such as mean, variance, or skewness) to be modeled either as a linear or additive function of covariates. Modeling by using GAMLSS is both flexible and robust, and it allows adding smoothing functions, such as cubic splines, penalized splines, and locally weighted regression, in order to improve the prediction power. Three different distributions included in GAMLSS implementation, all capable of modeling both skewness and kurtosis, were considered. These are the t-family Box-Cox Cole and Green distribution that models the median (mean), variance, and skewness of the outcome, the Box-Cox t distribution, and the Box-Cox Power Exponential distribution, both model kurtosis in addition to the median, variance, skewness, and kurtosis of the outcome.

Percentiles and Z-scores were calculated and extracted as described by Rigby and Stasinopoulos.^16^ The Analysis of Growth Data package was used to extract the coefficients of smoothing parameters. Growth curves based on these percentiles and Z-scores were generated accordingly and compared with WHO standards (see the following text).

The comparison of the Bahraini anthropometric indices of infants and young children with their counterparts in the WHO standards was conducted using the Bland-Altman (BA) method and mixed effects regression modeling as described in Giavarina.^17^ Briefly, for each anthropometric index (weight, length, and BMI), the agreement (difference) between the percentile or Z-score as calculated for the Bahraini subjects and the corresponding WHO standard was examined, separately, for each sex. The association was first established between the two corresponding data using the WHO standard as a reference or gold standard represented by the x-axis and the Bahraini data represented by the y-axis. In the BA method, the x-axis shows the main paired measurements, while the y-axis indicates the difference between the two paired methods (the bias), presented either in absolute units or as a percentage. The BA method also calculates the 95% limits of agreement (LOA) as the mean difference (2 SD or 1.96 SD), and the results are interrupted by the BA Index, defined as the percentage of the difference between the tested and the gold falling beyond the LOA. According to this method, a value of <5% indicates good agreement. In the mixed-effects regression methodology, each anthropometric was modeled as a response with age as the fixed effect and including the mother’s age and gestational age as sources of random effects. Finally, the result of the mixed effects was integrated with the BA plot.

## RESULTS

**Figure 1.**
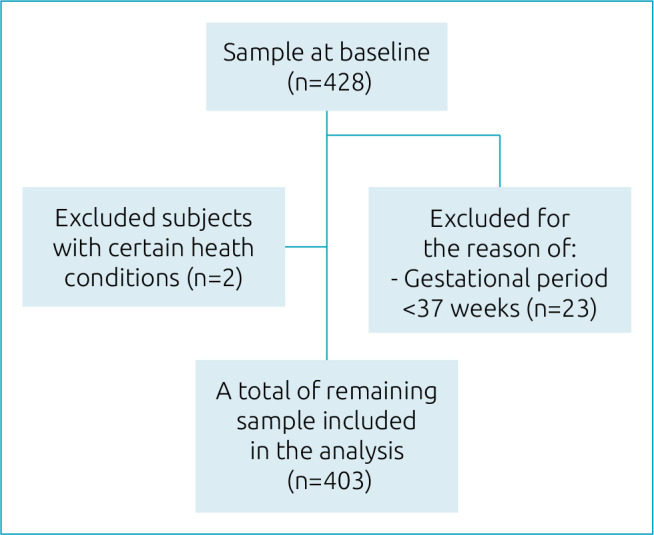
Flowchart representing the selection of the study samples.

This study included 428 children aged between 0 and 2 years selected from five governmental health centers, representing the targeted population of the study. In all, 25 of the originally selected subjects were excluded due to noncompliance with the inclusion criteria. A total of remaining 403 subjects (210 males and 193 females) were included in the analysis, as shown in Figure 1.

Vital data and nutritional status of infants in Bahrain in 2019 indicated that the total number of live births was 18.042 per 1,000. Of which, 2049 were premature births. In 2016, the mortality rate of children under 5 years of age was 7.6 deaths per 1,000 live births. The prevalence of stunting, wasting, underweight, overweight, and obesity was 4.4, 2.6, 2.4, 4.9, and 1.3%, respectively.^18^

Descriptive statistics for relevant demographic characteristics by sex are presented in Table 1. Sex within the sample size was roughly equally distributed between males (52%) and females (48%) (Table 1). Most of the subjects were a single child (97.4%), and the majority were normally delivered (vaginal: 79.9%). In terms of birth order, the second male child and the third female child were the most comprising 32.1 and 26.4%, respectively. The proportion of exclusively breastfed infants/children was 28.7% at 6 months. In contrast, 34.7% were not breastfed. Furthermore, 36.5% of children were having both breastfed and bottle-feeding at the same age.

**Table 1.** Demographic characteristics of subjects and subject’s parents according to sex.

	Males		Females		Total	
	n	%	n	%	n	%
Gender						
	210	52	193	48	403	100
Child history						
Single	205	98	191	99	396	98
Twin	5	2.4	2	1	7	1.7
Delivery type						
Vaginal	165	79	156	81	321	80
LSCS	44	21	37	19	81	20
Child rank						
First	42	20	48	25	90	23
Second	67	32	46	24	113	28
Third	52	25	51	26	103	26
Fourth	24	12	29	15	53	13
Fifth +	24	12	19	9.8	43	11
Gestational age						
	39	1.3	39	1.3	–	–
Mother’s age						
	30	5.7	30	5.9	–	–

LSCS: lower segment cesarean section.

Preliminary comparative post-hoc analysis using ANOVA showed that female and male groups significantly differed from each other at the 8-levels of age of 8 years with regard to weight and length (Table 2); hence, each sex was separately analyzed.

**Table 2.** Comparison between means of weight (kg), length (cm), and body mass index according to sex.

Age (months)		Weight				Length				BMI			
		F	M	Overall	p-value	F	M	Overall	p-value	F	M	Overall	p-value
Birth	Mean (SD)	3.021 (0.468)	3.195 (0.462)	3.112 (0.472)	<0.001	48.797 (2.217)	49.610 (2.267)	49.224 (2.277)	<0.001	12.652 (1.564)	12.982 (1.597)	12.825 (1.588)	0.032
2	Mean (SD)	4.957 (0.622)	5.447 (0.602)	5.214 (0.658)	<0.001	55.686 (2.073)	57.120 (2.250)	56.439 (2.280)	<0.001	15.965 (1.626)	16.684 (1.487)	16.342 (1.594)	<0.001
4	Mean (SD)	6.360 (0.764)	6.931 (0.799)	6.660 (0.832)	<0.001	61.264 (2.309)	62.844 (2.365)	62.093 (2.466)	<0.001	16.935 (1.751)	17.544 (1.725)	17.254 (1.762)	<0.001
6	Mean (SD)	7.411 (0.929)	7.980 (0.939)	7.710 (0.975)	<0.001	65.285 (2.503)	67.075 (2.310)	66.224 (2.562)	<0.001	17.387 (1.937)	17.727 (1.801)	17.565 (1.872)	0.061
9	Mean (SD)	8.500 (1.091)	9.187 (1.049)	8.861 (1.122)	<0.001	70.161 (2.713)	71.814 (2.807)	71.028 (2.881)	<0.001	17.247 (1.821)	17.805 (1.686)	17.540 (1.771)	0.001
12	Mean (SD)	9.425 (1.209)	10.032 (1.159)	9.743 (1.220)	<0.001	74.294 (3.182)	75.878 (2.953)	75.125 (3.161)	<0.001	17.070 (1.694)	17.403 (1.548)	17.245 (1.626)	0.035
18	Mean (SD)	10.792 (1.517)	11.498 (1.390)	11.162 (1.493)	<0.001	81.474 (3.365)	82.671 (3.343)	82.102 (3.403)	<0.001	16.245 (1.764)	16.810 (1.665)	16.542 (1.734)	<0.001
24	Mean (SD)	11.930 (1.619)	12.422 (1.401)	12.188 (1.527)	<0.001	87.250 (4.238)	88.100 (3.868)	87.696 (4.066)	0.031	15.709 (1.787)	16.016 (1.540)	15.870 (1.667)	0.058

BMI: body mass index; SD: standard deviation; F: female; M: male.

At birth, the mean weight of males and females was 3.2±0.4 and 3.01±0.4 kg, respectively. In all studied age levels, weight of male group was statistically significantly higher than that of female group with difference in weight ranging between 4 and 9% (Table 2).

Skewness is an inherited nature in growth measurements.^19^ In animals, body size distributions follow a right-skewed pattern.^19^ The LMS/GAMLSS method was used to model the measurement, smooth the model parameters, and finally estimate smoothed percentiles from the model parameters.

**Figure 2.**
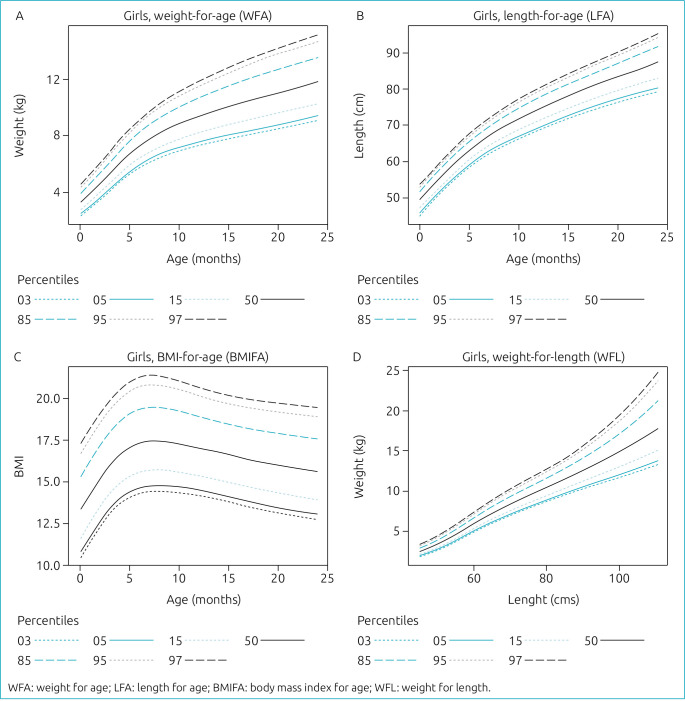
Percentiles for Bahraini female infants and young children aged 0–24 months. (A) is the plot for weight for age, (B) is for length for age, and (C) is for the body mass index for age, which shows a typical increase by age. (D) shows the increasing relation between weight for length.

**Figure 3.**
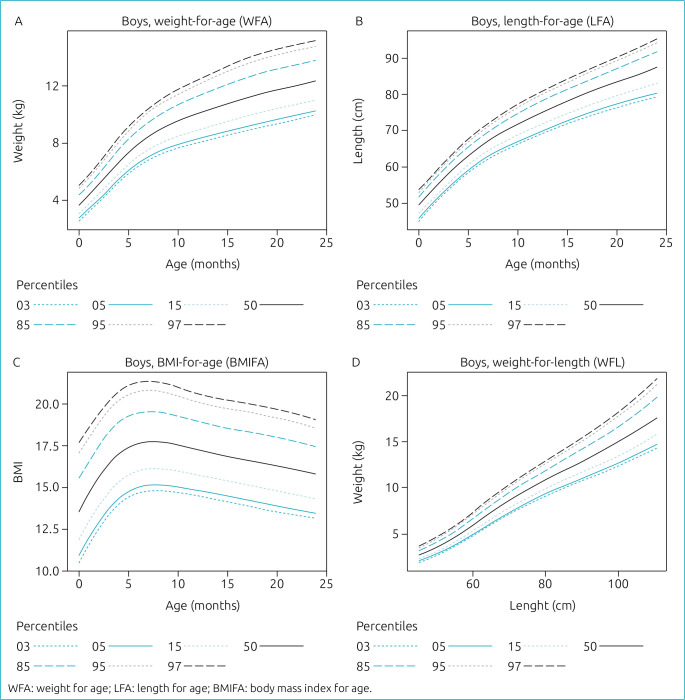
Percentiles for Bahraini male infants and young children aged 0–24 months. (A) shows the increasing plot for weight for age, (B) for length for age, and (C) for the body mass index for age. (D) shows the relation between weight for length.

The weight of females and males has shown a typical increase with age (Figures 2A and 3A). Similarly, the length of females and males also increased with the age of up to 2 years (Figures 2B and 3B). This considered a logical explanation for the increasing relation between weight for length for both females and males as shown in (Figures 2D and 3D). The BMI of females showed an increase up to around 6 months followed by a noticeable decrease from 6 months to 24 months (Figure 2C). On the contrary, the BMI of males showed a steep increase up to around 6 months, followed by a period of stability up to 10 months, and then decreased during the period up to 24 months, as shown in Figure 3C.

**Figure 4.**
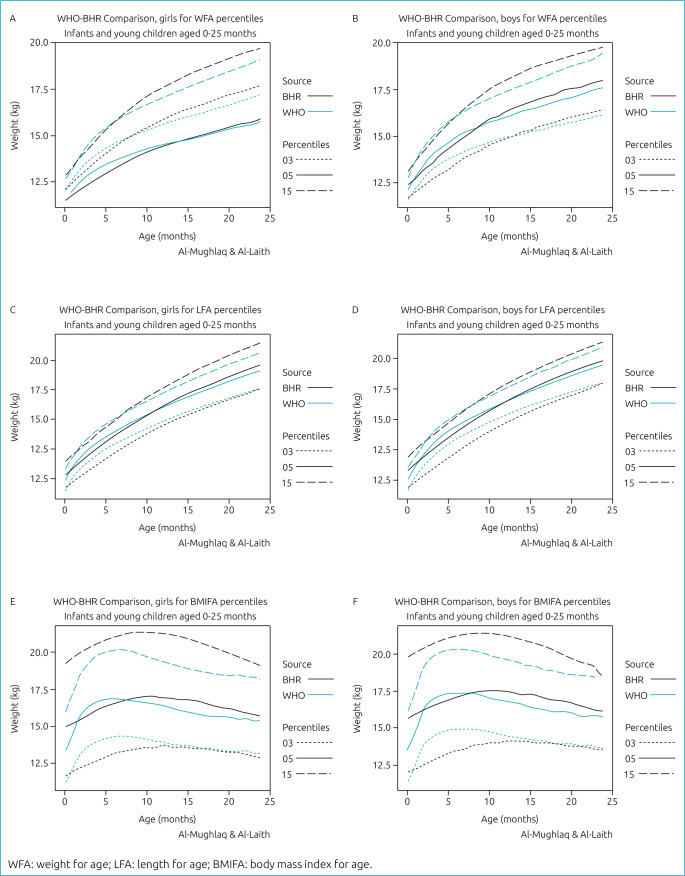
WHO-BHR comparison of girls and boys for weight for age (WFA), length for age (LFA), and BMI for age (BMIFA) percentiles.

Comparisons between the 3rd, 50th and 97th of weight, length and BMI centiles of Bahraini females and males and their counterparts in the WHO-MGRS standards, respectively, showed some variations in different age levels. Generally, at early infancy and at the three selected centiles, Bahraini females and males had lower weight than their MGRS counterparts. At higher age, this trend was reversed (Figure 4)

Bahraini females were shorter than MGRS females at earlier ages from 2 to 8 months but grew taller from 9 to 24 months at the 97th centile. Bahraini males were also shorter than MGRS males at the third centile at all ages except at birth. In contrast, they become taller at the 50th and 97th centiles; the exception was at the ages from 3 to 6 months (>0.5 SD) (Figure 4).

The BMI of Bahraini females was greater than that of MGRS females at the 97th centiles (SD >0.5). The BMI of Bahraini males at the third centiles was lower than of MGRS males at ages from 1 to 5 months (SD >0.5) and became larger than of those of MGRS at the 97th centiles at all ages (Figure 4).

Differences in weight and/or height may lead to differences in BMI. Although the differences in the third centile were less than the WHO-MGRS for both sexes, the increase in the BMI was obvious at the 50th centile starting from the 2nd month until the 20th month of age with an average of 0.4 with a similar trend in both sexes. At the 97th centile, the average BMI, increased over the WHO-MGRS in both sexes, was estimated to be 1.3, and the difference began to decrease toward the end of the 24 months of age. In general, the patterns of change in weight of both sexes at the three centiles were similar during the growth period 0 to 24 months (Figure 4).

Differences in weight between the two sexes began to appear starting from the 4th month and continue until the 20th month of age; the average difference in weight was estimated at 0.25, 0.4, and 0.8 kg at 3rd, 50th, and 97th centiles, respectively, with males being heavier than females.

## DISCUSSION

The present study is the first to examine the physical growth of infants and young children aged 0–24 months of both sexes using the GAMLSS/LMS method in the Kingdom of Bahrain. The study evidently demonstrated differences between both sexes and that the anthropometric measurements (weight, length, and BMI) of boys were slightly higher than girls. This is in line with findings previously reported in many countries such as in United Arab Emirates^9^ and Malaysia.^20^

The findings of this study also clearly indicate the difference between the WHO-MGRS standards and the Bahraini growth anthropometric measurements. In particular, the weight of girls and boys was heavier than their cohorts in MGRS. Similar findings were reported by Natale and Rajagopalan, who concluded that significant variations existed between ethnic and national groups in terms of weight, height, and other growth parameters worldwide.^7^ A similar study of Euro-growth references for length, weight, and body circumferences identified four 0.5 SD outliers among males and females.^21^

Regarding length (height), at the age from 0 to 24 months, Bahraini girls and boys were statistically shorter in the first 6 months of age than the MGRS standard and became taller later up to 2 years. This is in line with the findings of a German study that reported that the height means for males and females up to age 5 differ particularly at the 62nd and 60th MGRS centiles, respectively.^22^ These authors considered these differences sufficient to warrant the use of national growth charts rather than the MGRS charts in Germany.

In addition, Atladottir and Thorsdottir. measured children at birth and at 12 months in Iceland and found that the average length of all children was approximately two-thirds of an SD taller than the MGRS charts at these two time points.^23^ Furthermore, male and female infants up to the age of 1 year in Denmark were considered outliers in terms of length.^24^ Similarly, Fredriks et al. found that Moroccan infants among those living in the Netherlands were also outliers at age one.^25^

The BMI of both males and females included in the current study was significantly higher than MGRS standards at the 97th centiles in most age groups up to 2 years. Similarly, the BMI of US children was remarkably different from MGRS standards, partly reflecting obesity numbers in the US sample, and, probably as well, edge effects in the 2000 growth charts.26 Consequently, the estimates of overweight and obesity increased when the MGRS standards were used for US children. Furthermore, the significant difference in the BMI for age between the 2000 and 2006 WHO growth charts at the -2 SD and -3 SD resulted in a lower estimation of undernutrition.^26^

In contrast to the MGRS study, which considers only the growth of healthy breastfed children as a normative model for growth and development,^2^ in the current study, both breastfed and formula-fed children were considered for setting norms. Despite the fact that the WHO and most prominent pediatric societies advocate practicing exclusive breastfeeding for infants from birth to 6 months,^27^ the prevalence of exclusive breastfeeding remains low in many countries.^28^ Likewise, in Bahrain, the rates of exclusive breastfeeding among children under 6 months in 2014 were 30%,^18^ and dramatically decreased to 11% in 2019.^29^ This justifies the higher proportion of the selected children in this study were mostly fed with breast-milk substitutes.

The impact of breastfeeding on the linear growth of children has been examined by several studies. However, breastfed children tend to be smaller than their formula-fed cohorts.^30^ Furthermore, studies that examined the association between breastfeeding and obesity in early childhood found that breastfeeding is a significant protective factor against obesity, and nonbreastfed children were more likely to be obese than breastfed ones.^30,31^

In terms of length, breastfed children’s lengths were closer to local growth standards than to the WHO standards.^32^ This was supported by Tanaka et al., who found that Japanese breastfed children were below the MGRS mean at every age measured with at least a 0.5 SD difference. While the difference between formula-fed children’s means was either within 0.25 SD or not below 0.5 SD of the MGRS means.^33^

The MGRS-WHO charts were based on children who were living under conditions likely to achieve full growth potential.^6,12^ In particular, it included children from a diverse set of countries from different regions: Brazil, India, Ghana, Norway, Oman, and the USA.^34^ Besides this, the mothers of the selected children used for developing the standards were aged between 18 and 35 years and engaged in health-promoting practices, namely being healthy and being nonsmokers without any associated diseases during pregnancy.^34^ Due to a lack of information in the child’s health record, the present study does not consider the age and health condition of pregnant mothers during pregnancy in the inclusion criteria.

In view of these methodological differences between the present study and WHO-MGRS, it is very probable that the growth of these children may be deemed “normal” within the characteristics of the MGRS-WHO that describe the ideal growth pattern. Whereas the observation of the difference-magnitude dependency on the local curves that describes the typical growth patterns of Bahraini children may represent a deviation from what is considered healthy.

The comparison between the local and the WHO-MGRS curves could provide an evidence-based foundation for further studies in the same context. In addition, it provides a rationale for selecting growth references for objective evaluation of the child’s growth and health status, particularly in clinical and/or public healthcare services and epidemiology, as well as to screen for malnutrition at both collective and individual levels in Bahrain. In contrast, these findings predict that the health and nutritional status of both infants and mothers during pregnancy in Bahrain require further investigation.

The constructed charts presented in this study showed significant differences in comparison with the WHO-MGRS reference charts. Specifically, Bahraini children aged between 0 and 24 months of both sexes were taller and heavier than their cohorts in MGRS reference charts. Further longitudinal studies are needed for monitoring the growth pattern of children using body composition methods, adiposity markers, and determinant factors of growth to investigate this deviation from the WHO-MGRS.

## Data Availability

The database that originated the article is available with the corresponding author.
